# Increased Urine Excretion of Neutrophil Granule Cargo in Active Proliferative Lupus Nephritis

**DOI:** 10.34067/KID.0000000000000491

**Published:** 2024-07-02

**Authors:** Nicholas A. Shoctor, Makayla P. Brady, Kenneth R. McLeish, Rebecca R. Lightman, Leshaia Davis-Johnson, Conner Lynn, Anjali Dubbaka, Shweta Tandon, Michael W. Daniels, Madhavi J. Rane, Michelle T. Barati, Dawn J. Caster, David W. Powell

**Affiliations:** 1 Division of Nephrology and Hypertension, University of Louisville School of Medicine, Louisville, Kentucky; 2 Department of Biochemistry and Molecular Genetics, University of Louisville School of Medicine, Louisville, Kentucky; 3 University of Louisville School of Medicine, Louisville, Kentucky; 4 Department of Internal Medicine, University of Louisville School of Medicine, Louisville, Kentucky; 5 Department of Bioinformatics and Biostatistics, University of Louisville School of Public Health and Information Sciences, Louisville, Kentucky

**Keywords:** cell activation, immunology and pathology, lupus nephritis, lymphocytes

## Abstract

**Key Points:**

- Neutrophil degranulation participates in glomerular injury in proliferative lupus nephritis.
- Urine excretion of neutrophil granule proteins is a potential diagnostic for proliferative lupus nephritis.

**Background:**

Lupus nephritis (LN) occurs in more than half of patients with systemic lupus erythematosus, but the cellular and molecular events that contribute to LN are not clearly defined. We reported previously that neutrophil degranulation participates in glomerular injury in mouse models of acute LN. This study tests the *hypothesis* that glomerular recruitment and subsequent activation of neutrophils result in urine excretion of neutrophil granule constituents that are predictive of glomerular inflammation in proliferative LN.

**Methods:**

Urine and serum levels of 11 neutrophil granule proteins were measured by antibody-based array in patients with proliferative LN and healthy donors (HDs), and the results were confirmed by ELISA. Glomerular neutrophil accumulation was assessed in biopsies of patients with LN who contributed urine for granule cargo quantitation and normal kidney tissue by microscopy. Degranulation was measured by flow cytometry in neutrophils isolated from patients with LN and HD controls by cell surface granule markers CD63 (azurophilic), CC66b (specific), and CD35 (secretory). Nonparametric statistical analyses were performed and corrected for multiple comparisons.

**Results:**

Eight granule proteins (myeloperoxidase, neutrophil elastase, azurocidin, olfactomedin-4, lactoferrin, alpha-1-acid glycoprotein 1, matrix metalloproteinase 9, and cathelicidin) were significantly elevated in urine from patients with active proliferative LN by array and/or ELISA, whereas only neutrophil elastase was increased in LN serum. Urine excretion of alpha-1-acid glycoprotein 1 declined in patients who achieved remission. The majority of LN glomeruli contained ≥3 neutrophils. Basal levels of specific granule markers were increased in neutrophils from patients with LN compared with HD controls. Serum from patients with active LN stimulated specific and secretory, but not azurophilic granule, release by HD neutrophils.

**Conclusions:**

Circulating neutrophils in patients with LN are primed for enhanced degranulation. Glomerular recruitment of those primed neutrophils leads to release and urine excretion of neutrophil granule cargo that serves as a urine marker of active glomerular inflammation in proliferative LN.

## Visual Abstract

**Figure:**
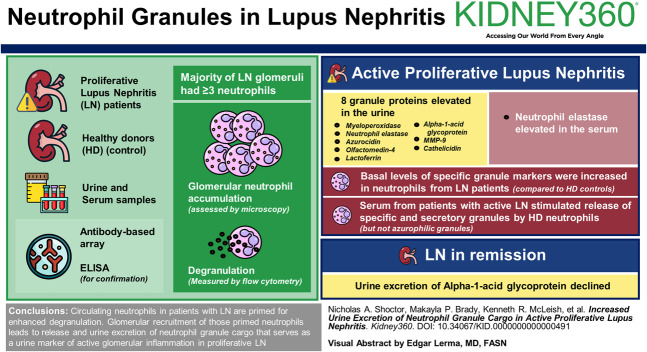

## Introduction

Systemic lupus erythematosus (SLE) is a complex autoimmune disease controlled by genetic, epigenetic, environmental, immune regulatory, and hormonal factors.^1,2^ Of the numerous organ systems affected, kidney involvement, termed lupus nephritis (LN), is a leading cause of morbidity and mortality in patients with SLE. Up to 50% of adults and 80% of children with SLE develop LN, and up to 20% of patients with LN progress to ESKD, requiring dialysis or transplantation.^3,4^ The renal histopathology, clinical presentation, course, and outcome of LN vary considerably. A classification system for renal histopathology identifies patients with the highest risk of developing kidney failure and is currently used to guide therapy.^5,6^ This classification system assigns biopsies to one of six classes: class 1, mesangial immune complex deposition without hypercellularity; class 2, mesangial hypercellularity; class 3, focal endocapillary/extracapillary hypercellularity (<50% of glomeruli); class 4, diffuse endocapillary/extracapillary hypercellularity (≥50% of glomeruli); class 5, membranous nephropathy (MGN); and class 6, glomerular and interstitial fibrosis.^7^ Patients with class 3 or 4 histopathology are at higher risk of developing renal failure, leading to the recommendation of treatment with corticosteroids and nonspecific immunosuppressives. A successful therapeutic response is defined by improvement in GFR and/or reduction in proteinuria.^3,4,6^ Follow-up or protocol biopsies, however, show that this assessment of therapeutic response can be misleading. Continued proteinuria may indicate inadequate response to therapy, conversion of histopathology from proliferative to membranous LN, or development of secondary focal segmental glomerulosclerosis.^8^ In addition, biopsies from patients with clinical resolution of proteinuria often show persistent inflammation,^9,10^ and up to a third of patients with remission of proteinuria show progressive loss of GFR.^8^

The limitations, cost, and risks of repeated kidney biopsies have led to research focused on finding reliable urine biomarkers that better correlate with histologic changes, disease activity, and response to therapy. Ideally, those biomarkers would also elucidate pathophysiologic mechanisms operating in individual patients, allowing a personalized approach to management. Although a number of urine biomarkers associated with LN have been identified, including IL-16, TGF-*β*, neutrophil gelatinase-associated lipocalin, monocyte chemoattractant protein-1, kidney injury molecule-1, adiponectin, hemopexin, ceruloplasmin, CXCL1, CXCL10, and the presence of specific immune cells,^11–15^ none are routinely used in clinical practice.

Neutrophil dysregulation in SLE is proposed to contribute to autoantibody development and organ dysfunction.^16–18^ Evidence supporting neutrophil contribution to LN includes neutrophil recruitment to glomeruli, the presence of neutrophil extracellular traps (NETs) within glomeruli, a neutrophil activation signature on gene profiling of blood and kidney, and presence of neutrophils in the urine.^19–26^ All of the mechanisms by which neutrophils kill invading microbes, including generation of toxic oxygen radicals, release of toxic chemicals from preformed granules, and NET formation, are proposed to induce tissue injury in SLE.^16–18^ We previously reported that inhibition of neutrophil degranulation prevented endothelial cell damage, podocyte effacement and loss, and proteinuria in immune complex-mediated GN in wild-type and LN-prone mice.^27,28^ In addition, coculture of neutrophil granule constituents with podocytes induced disordered cytoskeletal organization.^28^ Transcriptional analysis of blood leukocytes identified neutrophil granule constituents within activation signatures associated with LN.^23,25,26^ Those data suggest that glomerular neutrophil recruitment and subsequent release of granule constituents contribute to glomerular injury in LN. This study examined the *hypothesis* that urine excretion of neutrophil granule constituents predicts glomerular inflammation in proliferative LN.

## Methods

### Human Studies

The University of Louisville Institutional Review Board (IRB) approved all patient and normal individual sample collections and studies (IRB 01.0536 and IRB 96.0191). All patients with LN had biopsy-proven LN and positive antinuclear and/or anti–double-stranded DNA antibodies at diagnosis. All patient samples used for the RayBiotech Array were obtained from patients in active disease at the date of collection (Supplemental Table 1). Active disease state for LN was defined as urine protein to creatinine ratio (UPCR) exceeding 500 mg/g at the time of collection. Samples used for validation by ELISA (Supplemental Table 2) to compare active LN versus remission (Supplemental Table 3) and to assess neutrophil degranulation (Supplemental Table 4) were obtained from patients during an outpatient clinic visit. Healthy donors (HDs) had no underlying health conditions, were not on any medications, and were not anemic on day of collection. All nephrotic control patients had a UPCR exceeding 3.5 g/g at the time of collection.

### Neutrophil Isolation

Whole blood was collected from patients and HDs. Serum was obtained by centrifugation at 800×*g*, aliquoted, and stored at −80°C. Neutrophils were isolated from whole blood by plasma-percoll density gradient centrifugation, as previously described.^29,30^

### Custom Protein Array

An antibody-based array (Ray Biotech) was constructed to measure 11 known neutrophil granule proteins (lactoferrin, cathelicidin, gelatinase matrix metalloproteinase 9 [MMP-9], olfactomedin-4, alpha-1-acid glycoprotein 1 [*α*-1AG], neutrophil elastase, azurocidin, myeloperoxidase [MPO], catalase, ficolin-1, and proteinase 3) and two proteins associated with NETs (peptidyl arginine deiminase 4 [PADI4] and citrullinated histone H3.3). Urine protein values were normalized to urine creatinine levels.

### ELISA

*α*-1AG (Abcam-Waltham, MA), MMP-9 (R&D Systems-Minneapolis, MN), neutrophil elastase (Abcam), MPO (Abcam) azurocidin (Abcam), and lactoferrin (Abcam) were quantitated by ELISA according to the manufacturers' instructions. The measured protein concentrations were normalized to urine creatinine concentrations (mg/ml) from each sample.

### Degranulation

Neutrophil plasma membrane expression of CD35, CD66b, and CD63 were measured *via* flow cytometry, as previously described.^30,31^ For CD63, an incubation with latrunculin, an inhibitor of actin polymerization, was required for the *in vitro* release of azurophilic granules.

### Immunofluorescence and Confocal Microscopy

Formalin-fixed paraffin-embedded renal biopsy sections from patients with class 4 and 4/5 LN were used. Kidney wedge samples (Formalin-fixed paraffin-embedded) from kidneys obtained from deceased donors unsuitable for transplantation (courtesy of Kentucky Organ Donor Affiliates; approved by the University of Louisville Human Studies Committee) served as controls.

Sections were cleared of paraffin, rehydrated in graded ethanol, and subject to antigen retrieval with pH 6.0/citrate buffer. Sections were blocked in 5% horse serum/tween-tris-buffered saline and then incubated with anti-MPO (1:200; Abcam 9535) in 1% BSA/tween-tris-buffered saline, overnight at 4°C, followed by secondary antibody incubation with donkey anti-rabbit Alexa-Fluor 546. Next, sections were incubated with allophycocyanin-conjugated anti-CD66b (1:50; miltenyi biotec 130-117-692) for 15 minutes, and nuclei stained with 4’,6-diamidino-2-phenylindole. Images were acquired using an Olympus Fluoview FV-1000 confocal coupled to an Olympus 1X81 inverted microscope, 60× objective, and FV-10 ASW 2.1 software. A multichannel scanning configuration with sequential line scanning was setup for acquisition of 4’,6-diamidino-2-phenylindole, Alexa-Fluor 546, and allophycocyanin. Each setting was tested against secondary antibody-alone control sections to ensure exclusion of nonspecific emission.

### Combined MPO Immunohistochemistry and PAS Staining

Renal biopsy and wedge sections were processed for antigen retrieval and incubated with anti-MPO antibody as with immunofluorescence staining. All sections, including negative controls, were then incubated with biotinylated goat-anti-rabbit secondary antibody (Vector Labs) for 30 minutes, followed by incubation in avidin:biotin enzyme complex (Vectastain Elite ABC kit, Vector Labs) for 30 minutes. Proteins were detected after color development using 3,3′-diaminobenzidine as substrate (Vector Labs). Sections were then subject to PAS (periodic acid Schiff; ScyTek) staining by incubation in periodic acid for 4 minutes and Schiff reagent for 3 minutes. Images were acquired with a Q Color 5 camera attached to an Olympus BX51 microscope using Image-Pro software. MPO staining was tabulated by counting MPO-stained neutrophils in glomeruli. Total cells/total glomeruli analyzed was calculated for each section.

### Statistical Analyses

Statistical analyses were performed using GraphPad Prism 10.2.1 and R 4.3.2. Comparisons of two independent groups were analyzed using Mann–Whitney tests with *post hoc* Bonferroni correction for multiple comparisons. Comparisons of more than two independent groups were analyzed using Kruskal–Wallis tests with *post hoc* corrected Dunn tests for multiple comparisons. Paired LN patient samples (*n*=15) were compared using a two-tailed Wilcoxon matched pairs signed rank test. Data obtained by microscopy were compared using a series of Mann–Whitney tests. The threshold for statistical significance was 0.05. All bar graphs display means and SD. Logistic regression analysis was performed using the glm function in R, and a receiver operating characteristic curve was subsequently generated using the pROC R package to determine how well a given protein value can predict a patient's disease status (active or inactive LN). The area under the curve was obtained using the pROC R package. The sensitivity, specificity, and optimal cutoff values were determined using the cutpointr R package to maximize the sum of sensitivity and specificity.

## Results

### Urine Excretion of Neutrophil Granule Proteins in Active Proliferative LN

**Figure 1:**
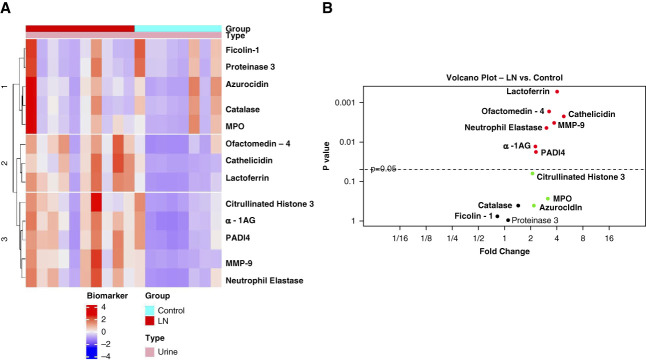
**Urine excretion of neutrophil granule proteins is enhanced in LN.** Urine protein concentrations of 11 granule proteins and two NET-associated proteins were measured by custom antibody-based array (RayBiotech) and normalized to urinary creatinine concentrations in ten patients with LN and eight HDs. (A) The scaled and centered data were plotted as a heatmap in which the different colors represent biomarker expression levels. The biomarkers and samples were then subjected to hierarchical clustering based on Euclidean distance. (B) Volcano plot where each point represents one protein. The protein values were summarized by mean and SD or median with minimum and maximum responses across the groups. The fold change between groups was calculated as the ratio of the mean or median. If a protein met or did not meet normality criteria across the group, the significance of expression difference was evaluated by the paired *t* test or signed-rank test, respectively. Proteins with FDR <0.05 were considered as differentially expressed. *α*-1AG, alpha-1-acid glycoprotein 1; FDR, false discovery rate; LN, lupus nephritis; MMP-9, matrix metalloproteinase 9; MPO, myeloperoxidase; NET, neutrophil extracellular trap; PADI4, peptidyl arginine deiminase 4.

To evaluate urinary excretion of neutrophil granule proteins in patients with active proliferative LN, a unique, antibody-based, commercial array was designed to quantify granule proteins highly expressed in three neutrophil granule subsets: azurophilic granules (proteinase 3, azurocidin, MPO, catalase, and neutrophil elastase), specific granules (lactoferrin, olfactomedin-4, and *α*-1AG), and gelatinase granules (MMP-9, ficolin-1, and cathelicidin).^32,33^ The array was used to quantify urine and serum granule protein content from ten patients with proliferative LN with active disease and eight HDs. Urine excretion of granule proteins was normalized to urine creatinine levels. A summary of demographics and clinical information for those ten patients with LN is shown in Supplemental Table 1. Figure 1 shows that neutrophil elastase (*P* = 0.004), olfactomedin-4 (*P* = 0.002), lactoferrin (*P* = 0.0005), MMP-9 (*P* = 0.003), *α*-1AG (*P* = 0.01), and cathelicidin (*P* = 0.002) were significantly increased in the urine from patients with LN, compared with HDs.

As NETs containing granule constituents are present within glomeruli in LN,^34^ the possibility that urine excretion of granule proteins resulted from NETosis, not degranulation, was evaluated by measuring urine concentration of PADI4 and citrullinated histones. PADI4 is a component of gelatinase granules,^32,33^ is involved in NET formation, and is present on NETs formed after A23187, but not PMA, stimulation.^35^ Citrullinated histones are ubiquitous components of NETs.^35^ PADI4 was significantly elevated in LN urine, whereas no significant increase in citrullinated H3.3 was observed. Expression of PADI4 in gelatinase granules and the absence of increased urinary excretion of citrullinated histones suggest that increased urinary excretion of granule proteins resulted from neutrophil degranulation, not NET formation.

To validate the results from the array assay, an ELISA for representative constituents of each granule subset was performed with a second cohort of patients with active proliferative LN and HDs (Supplemental Table 2). To assess whether the increase in urine neutrophil granule proteins was nonspecifically related to proteinuria, we also evaluated these proteins in nephrotic (UPCR >3.5 g/g) glomerular disease controls including pure class 5 LN (avg. UPCR 6.199 g/g), minimal change disease (avg. UPCR 6.887 g/g), and primary MGN (avg. UPCR 10.478 g/g). The validation candidates included neutrophil elastase, *α*-1AG, MMP-9, and lactoferrin that were significantly increase in LN patient samples from the array analysis. MPO and azurocidin were also included for ELISA validation because they had a >2-fold increase in urinary excretion by patients with LN (Figure 1B).

**Figure 2:**
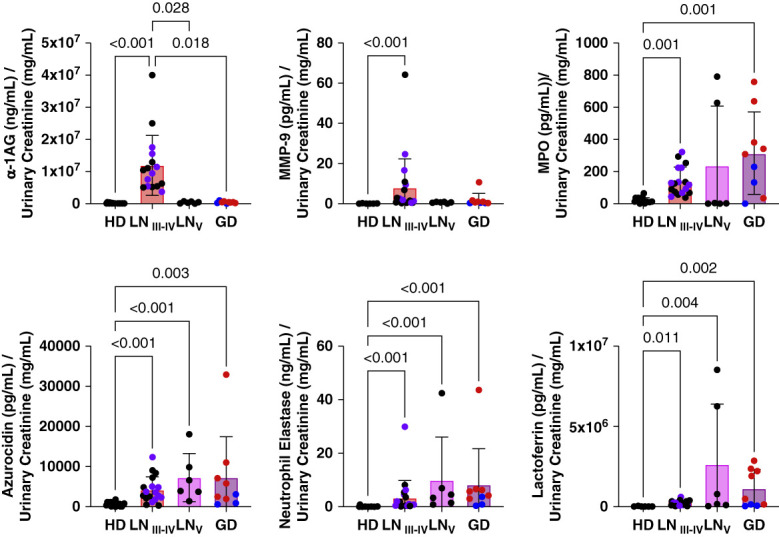
**Enhanced urine excretion of granule proteins is associated with LN activity.** Enhanced excretion of granule proteins were validated and compared in urine from patients with proliferative LN (LN 3–4), class 5 LN (LN 5), MCD, and primary MGN (other GD) and HDs by ELISA with normalization to urine creatinine concentrations. Azurophilic granule constituents measured included MPO (*n*=16 HD, *n*=18 LN 2–4, *n*=6 LN 5, *n*=9 GD), neutrophil elastase (*n*=16 HD, *n*=21 LN 2–4, *n*=6 LN 5, *n*=9 GD), and azurocidin (*n*=16 HD, *n*=18 LN 2–4, *n*=6 LN 5, *n*=9 GD). Specific granule constituents measured were *α*-1AG (*n*=15 HD, *n*=16 LN 2–4, *n*=6 LN 5, *n*=9 GD) and lactoferrin (*n*=6 HD, *n*=21 LN 2–4, *n*=6 LN 5, *n*=9 GD). The gelatinase granule constituent measured was MMP-9 (*n*=6 HD, *n*=21 LN I2–4, *n*=6 LN 5, *n*=9 GD). *P* values are shown for comparisons with statistical significance. MGN (red) and MCD (blue) results were include together as other GDs. In the proliferative LN group, purple represents patients with crescentic glomeruli on kidney biopsy. Enhanced granule protein excretion of granule proteins was validated in a separate patient cohort by ELISA with normalization to urine creatinine concentrations. The data were statistically analyzed using a series of Kruskal–Wallis tests with *post hoc*–corrected Dunn tests for multiple comparisons. Data were represented as mean±SD. GD, glomerular disease; HD, healthy donor; MCD, minimal change disease; MGN, membranous nephropathy.

The results indicate that urine *α*-1AG, MMP-9, MPO, azurocidin, neutrophil elastase, and lactoferrin were increased in patients with active proliferative LN compared with HDs (Figure 2). Patients with urinary *α*-1AG were increased in active proliferative LN compared with nephrotic disease controls. The mean urine *α*-1AG level in patients with active proliferative LN was higher than all nephrotic disease controls. The mean urine MMP-9 in patients with active proliferative LN was higher than 93% of patients with nephrotic disease. MPO, azurocidin, neutrophil elastase, and lactoferrin urine levels were elevated in active proliferative LN and nephrotic disease controls compared with HDs, but the variation in values suggest that the elevation was not in direct correlation to proteinuria.

### Specific Granule Protein *α*-1AG as a Urinary Biomarker of LN

**Figure 3:**
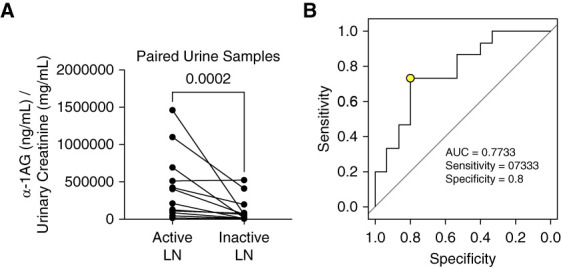
**Urine levels of specific granule protein *α*-1AG decease with LN remission.** (A) *α*-1AG concentrations in paired urine samples from patients with proliferative LN during active disease and during remission (inactive LN) were measured by ELISA and normalized to urine creatinine concentrations. The normalized values were statistically analyzed using a Wilcoxon matched-pairs signed rank test (*n*=15 paired samples) and *P* value shown. (B) A ROC curve was generated using the data from (A) to determine how well a given protein value can predict a patient's disease status (active or inactive LN). The optimal cutoff value between disease states was marked by a yellow circle. ROC, receiver operating characteristic.

Of the granule proteins that were validated in Figure 2, *α*-1AG had the highest average urine concentrations in the proliferative LN cohort and was significantly higher than levels in urine from class 5 LN and minimal change disease and MGN patients. *α*-1AG is also implicated as a candidate urinary marker for diagnosis of LN in patients with SLE.^36–38^ Thus, to examine the relationship of elevated urine *α*-1AG excretion to LN disease activity, *α*-1AG ELISA was performed with paired urine samples obtained from a separate cohort of 15 patients with proliferative LN comparing active LN (UPCR >0.5 g/g) and remission (UPCR <0.5 g/g). Clinical characteristics of those patients are shown in Supplemental Table 3. Figure 3A shows that mean urine excretion of *α*-1AG was significantly lower during remission in this LN cohort, suggesting utility of *α*-1AG as a urinary marker for monitoring activity of proliferative LN. The receiver operating characteristic curve for *α*-1AG in Figure 3 shows an area under the curve of 0.7733, suggesting urine *α*-1AG excretion is a candidate biomarker for active versus inactive LN. The optimal cutoff value between disease states was 89.23 *µ*g/ml (normalized to urinary creatinine).

### Neutrophil Degranulation Is Enhanced in LN Patients

**Figure 4:**
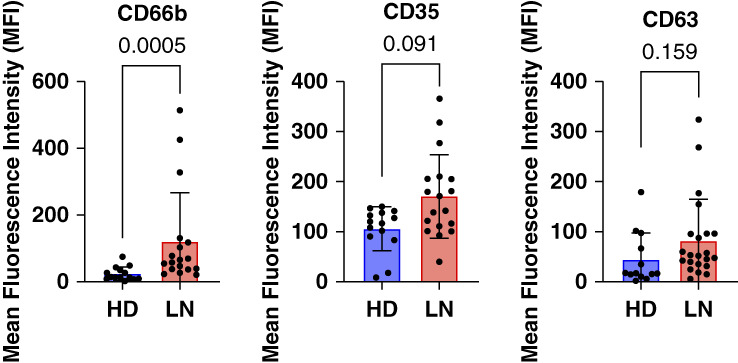
**Neutrophil degranulation is enhanced in patients with LN.** Neutrophil degranulation was assessed by untreated/basal cell surface expression of markers for secretory vesicles (CD35; *n*=15 LN, *n*=14 control), specific granules (CD66; *n*=15 LN, *n*=13 control), and azuophilic granules (CD63; *n*=22 LN, *n*=13 control) by flow cytometry. Statistical analysis comparing the two independent groups was performed by Mann–Whitney tests with *post hoc* Bonferroni correction for multiple comparisons and *P* values shown. Data were represented as mean±SD. MFI, mean fluorescence intensity.

Glomerular filtration barrier dysfunction in LN leads to leakage of plasma proteins into the urine. To evaluate the possibility that systemic neutrophil degranulation could result in increased urinary excretion, degranulation by peripheral blood neutrophils from patients with LN and serum levels of neutrophil granule constituents were determined. The clinical characteristics and demographics of patients providing blood for degranulation assays are shown in Supplemental Table 4. *In vivo* neutrophil degranulation was estimated from basal levels of plasma membrane expression of markers of secretory vesicles (CD35), specific granules (CD66b), and azurophilic granules (CD63) on isolated peripheral blood neutrophils. Figure 4 shows that neutrophils from patients with LN have a significantly increased plasma membrane expression of CD66b, compared with HDs, whereas basal CD35 and CD63 expression was higher in neutrophils from patients with LN but not statistically different.

**Figure 5:**
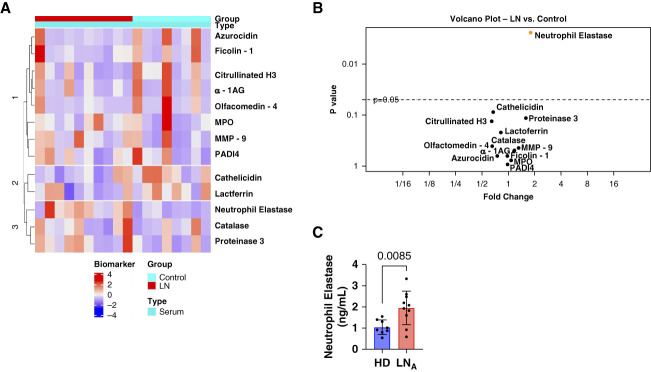
**Serum concentration of granule proteins in LN.** (A) Serum from the same ten patients with LN and eight HDs was examined for expression of 11 granule proteins and two NET-associated proteins by custom antibody-based array (RayBiotech). The scaled and centered data were plotted as a heatmap in which the different colors represent biomarker expression levels. The proteins and samples were then subjected to hierarchical clustering based on Euclidean distance. (B) Volcano plot where each point represents one biomarker. The protein values were summarized by its mean and SD or median with minimum and maximum responses across the groups. The fold change between groups was calculated as the ratio of the mean or median. If a protein met or did not meet normality criteria across the group, the significance of expression difference was evaluated by the paired *t* test or signed-rank test, respectively. Proteins with FDR <0.05 were considered as differentially expressed. (C) ELISA validation of serum neutrophil elastase (*n*=10 active LN, *n*=6 control). LN_A_ represents active LN. The data were analyzed using a Mann–Whitney test. Data were represented as mean±SD.

Serum levels of all 11 granule constituents measured in urine were quantified from the same ten patients with LN and 8 health donors, using the antibody-based array. Figure 5, A and B show that, despite variability in serum levels in both patients and HDs, only neutrophil elastase was significantly increased in the serum of patients with LN (*P* = 0.002). This elevated serum neutrophil elastase was confirmed by ELISA (Figure 5C). Although neutrophil elastase is a constituent of azurophilic granules, increased urine excretion of other azurophilic granule constituents (MPO and azurocidin) occurred in the absence of elevated serum levels. In addition, constituents of specific granules (lactoferrin and *α*-1AG) and gelatinase granules (MMP-9) showed increased urine excretion in the absence of elevated serum levels.

**Figure 6:**
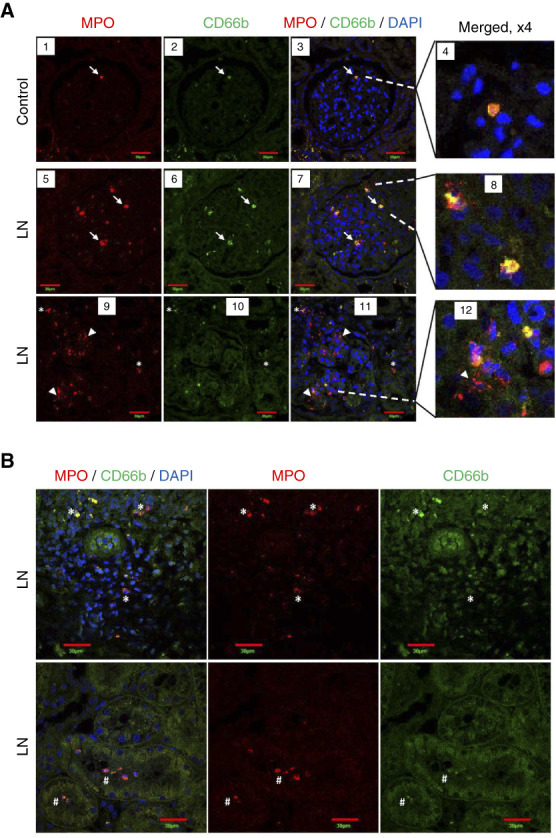
**Neutrophils in kidneys of patients with LN and controls.** Kidneys from controls and patients with LN were co-immunostained for MPO (red staining) and CD66b (green staining). Nuclei were stained with DAPI. (A) Glomerulus from a control (panels 1–4) shows an intact PMN (white arrow) expressing MPO and CD66b (colocalization shown in yellow). Glomeruli from patients with LN also contain intact PMNs (panels 5–8; white arrows) with MPO and CD66b colocalized within cells, as well as areas with dispersed/diffuse MPO staining, suggestive of extracellular localization (panels 9–12; white arrowheads). (B) Neutrophils positive for MPO and CD66b staining are present in the interstitial regions (white asterisk, top row) and inside tubule lumen (white pound sign, bottom row) in kidneys of patients with LN. *n*=4/group. Single plane confocal images are shown. Scale bars: 30 *µ*m. DAPI, 4’,6-diamidino-2-phenylindole; PMN, polymorphonuclear neutrophil.

Taken together, our results suggest that increased urinary excretion of granule cargo in patients with active class 3 or 4 LN is likely due to degranulation by neutrophils recruited to the kidney, rather than filtration of granule cargo release by circulating neutrophils. To test this postulate, glomerular recruitment and localization of neutrophils in LN was determined in kidney biopsies from four patients with class 4 and 4/5 LN who contributed urine for granule cargo quantitation and from kidney sections from four patients without SLE for which kidneys were unsuitable for transplantation. Neutrophils were identified by colocalization of immunostaining for CD66b and MPO by confocal microscopy. In control kidney sections, intact neutrophils were observed within glomerular capillaries (Figure 6A). Glomeruli from patients with LN also contain intact polymorphonuclear neutrophils (Figure 6, panels 5–8; white arrows) with MPO and CD66b colocalized within cells, as well as areas with dispersed/diffuse MPO staining, suggestive of extracellular localization and neutrophil degranulation (Figure 6, panels 9–12; white arrowheads). In LN, most glomeruli demonstrated >3 neutrophils (Figure 6A). In addition, neutrophils were observed in the tubular lumen in biopsies of patients with LN (Figure 6B).

**Figure 7:**
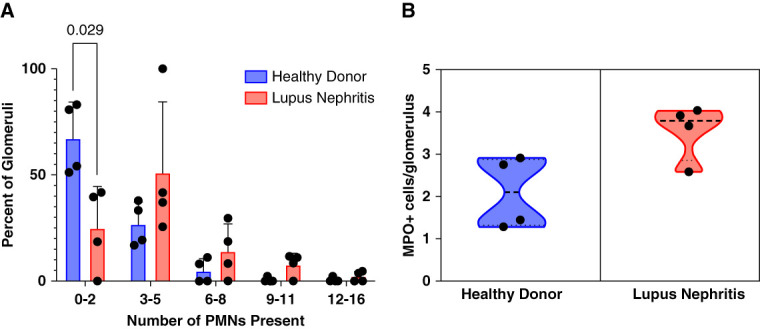
**Glomerular neutrophil recruitment in LN**. Kidney sections from patients with LN and controls were immunohistochemically stained for MPO. Total number of glomeruli in each section was counted and number of MPO-stained neutrophils in each glomerulus. (A) Percentage of glomeruli with different range of neutrophil numbers/glomerulus are shown. Bar graph represents average for each group (control or LN) with glomeruli containing neutrophils in each range (0–2, 3–5, 6–8, 9–11, 12–16), ±SD *n*=4/group. Data were analyzed using a series of Mann–Whitney tests. (B) Average number of neutrophils/glomerulus in control and LN sections. Ratio of total number of neutrophils/total number of glomeruli for each sample was calculated and averaged for each group. Data are displayed as averages±SD with *n*=4 per group.

To determine number and localization of neutrophils in glomeruli and correlation to histopathology associated with LN, kidney sections from patients with LN and controls were immunohistochemically stained for MPO, followed by PAS staining of the sections to define glomerular structure and pathology. A total of 191 and 85 glomeruli were analyzed in sections from controls and patients with LN, respectively. The majority (75%) of glomeruli from patients with LN contained three or more neutrophils, whereas nearly 67.2% of glomeruli from controls had ≤2 neutrophils (Figure 7A), suggesting increased neutrophil recruitment to glomeruli with LN. The average number of neutrophils/glomerulus in glomeruli from patients with LN and controls was 3.57±0.34 and 2.1±0.43, respectively (Figure 7B).

**Figure 8:**
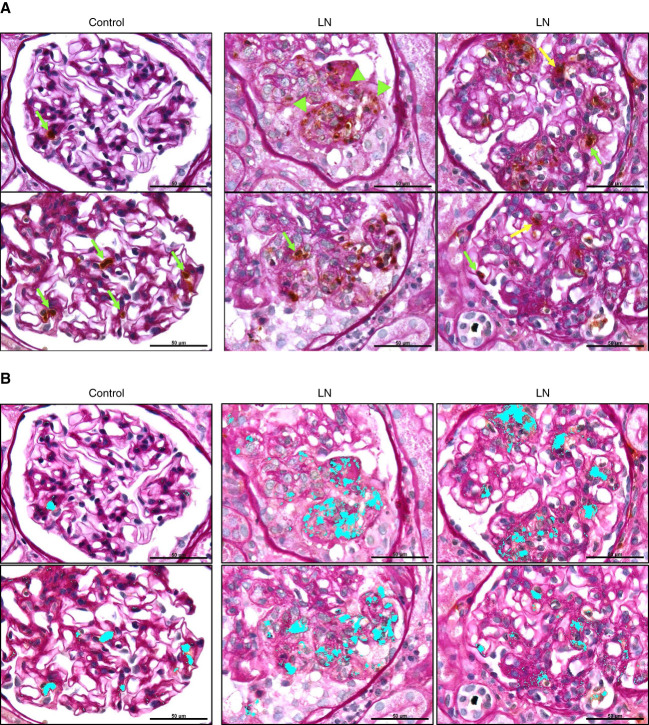
**Localization of neutrophils and pattern of MPO staining within glomeruli of patients with LN and controls.** To correlate neutrophil localization in glomeruli and MPO staining with glomerular histology, kidney sections from patients with LN and controls were immunohistochemically stained for MPO, followed by PAS staining of the section to define glomerular structure and pathology. (A) Glomeruli from controls show intact neutrophils (brown cells) contained within glomerular capillary loops (green arrows). Glomeruli from patients with LN contain intact neutrophils in capillary loops (green arrows) and the mesangial matrix or transmigrated from capillary lumen (yellow arrow). Glomeruli from patients with LN show diffuse/extracellular MPO staining around capillary loops or the mesangial matrix (green arrowheads). (B) To more clearly show the pattern of MPO staining in glomeruli, brown MPO staining was detected with Image Pro software in the same images as shown in (A), and the detected MPO staining area is highlighted in Cyan. Images are representative of *n*=4/group. Original magnification, 100×. Scale bar: 50 *µ*m. PAS, periodic acid–Schiff.

As shown in Figure 8A, glomeruli from controls show intact neutrophils (brown cells) contained within glomerular capillary loops (green arrows). Glomeruli from patients with LN also contain intact neutrophils in capillary loops (green arrows) and extracapillary and transmigrated neutrophils in the mesangial matrix (yellow arrow). Furthermore, similar to findings from immunofluorescence staining and confocal microscopy, glomeruli from patients with LN show diffuse/extracellular MPO staining around capillary loops or in the mesangial matrix (Figure 8A, green arrowheads), indicative of activation and degranulation. To more clearly show the pattern of MPO staining in glomeruli, software (Image Pro) detection of brown MPO staining was used in the same images as shown in Figure 8A, and the detected area is highlighted in Cyan (Figure 8B). These images more clearly show MPO staining limited to intact neutrophils in glomeruli of controls, whereas in glomeruli from patients with LN MPO staining is contained within intact cells and dispersed to extracellular compartments, suggesting degranulation. These data demonstrate glomerular neutrophil recruitment, transmigration, and degranulation in LN.

**Figure 9:**
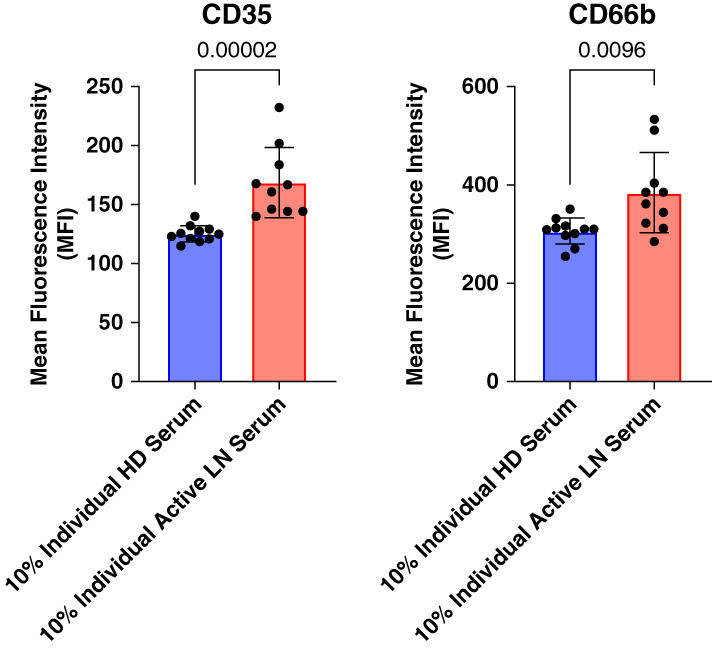
**Neutrophil degranulation is stimulated by LN sera.** Neutrophils isolated from HDs were incubated with sera from 11 patients with LN and nine HDs. Degranulation was measured as plasma membrane expression of CD35 for secretory vesicles and CD66b for specific granules. Statistical analyses for comparison of more than two independent groups were performed by Kruskal–Wallis tests with *post hoc*–corrected Dunn tests and *P* values shown. Data are displayed as averages±SD.

The enhanced degranulation of specific granules by peripheral blood neutrophils in patients with LN shown in Figure 4 suggested the presence of a neutrophil activating agent in the circulation. To evaluate this possibility, CD35, CD66b, and CD63 expression was measured in neutrophils from HDs incubated with sera from patients with proliferative LN with active disease (*n*=10) or from HDs (*n*=10). Figure 9 shows that sera from patients with LN stimulated a significant increase in CD35 and CD66b compared with incubation with sera from HDs. No differential effect of LN sera on CD63 expression was observed (data not included). These results are consistent with enhanced basal CD66b expression in patients with LN versus healthy control neutrophils as shown in Figure 4. These data suggest that circulating factors present in patients with LN stimulate a limited, low-level neutrophil degranulation that leads to enhanced neutrophil degranulation on exposure to immune complexes within glomeruli.

## Discussion

Although a role for neutrophils in LN has been questioned,^39^ increasing evidence suggests neutrophils are involved in glomerular injury in this disease. That evidence includes presence of neutrophils in glomeruli from LN biopsies by histology and gene expression,^19–22^ presence of neutrophils in the urine of patients with active LN,^14^ association of active neutrophil gene signature in the blood of patients with active LN,^23–26^ urine excretion of some neutrophil constituents, and glomerular injury mediated by neutrophil granule cargo in a mouse model of acute LN.^27,28,40–44^ The major finding of this study is that glomerular recruitment of activated neutrophils in active class 3 and 4 human LN is associated with urine excretion of neutrophil granule cargo. The absence of elevated serum levels of granule cargo supports intraglomerular neutrophil degranulation as the source of urine excretion of granule constituents.

During maturation in the bone marrow, neutrophils acquire four types of secretory granules. Three of those granule subsets, azurophilic (primary), specific (secondary), and gelatinase (tertiary) granules, contain cargo determined by protein synthesis of constituents at the time of granule formation. Proteins highly expressed in azurophilic granules include proteinase 3, azurocidin, MPO, catalase, neutrophil elastase, and tetraspanin CD63; specific granules include lactoferrin, olfactomedin-4, *α*-1AG, and carcinoembryonic antigen-related cell adhesion molecule 8 (CD66b); and gelatinase granules include MMP-9, ficolin-1, and cathelicidin.^32,33^ The fourth subset, secretory vesicles, is formed by endocytosis late in maturation and expresses numerous immune regulatory proteins, including complement receptor 1 (CD35), that are incorporated into the plasma membrane on exocytosis.^30,31^

The antibody-based array used in this study was designed to screen urine for excretion of representative cargo from azurophilic, specific, and gelatinase granules. The array results and ELISA validations show enhanced urine excretion cargo from all three granule subsets in patients with active proliferative LN, compared with HDs. There is also variability in levels of the candidate protein with regard to the different groups and no clear correlation with proteinuria. Thus, excretion of neutrophil granule cargo is not simply because of loss of filtration barrier leading to proteinuria and the results support specificity of granule cargo excretion to proliferative and/or class 5 LN.

Of note, reports show that patients with urine tract infections (UTIs) have evaluated urine levels of neutrophil proteins and that UTI is common in patients with LN receiving immunosuppressive medicine.^45,46^ To address this potential confounding factor, patients with known UTI at time samples collection were excluded from our analyses. From our list of validated candidates, *α*-1AG is a specific granule protein that is implicated as a candidate urinary diagnostic marker of LN in patients with SLE.^36,38,47^ We show that decreased urine levels *α*-1AG are significantly associated with clinical remission in our proliferative LN cohort, suggesting its additional use as a urinary marker for monitoring activity of proliferative LN. By array and ELISA, neutrophil elastase was the only granule protein significantly increased in serum from patients with LN, indicating that enhanced urine excretion of granule cargo is not a product of increased permeability of the glomerular filtration barrier. Taken together, we conclude that neutrophil recruitment into glomeruli during active proliferative LN leads to intraglomerular release of granule cargo which can be detected by enhanced urine excretion.

Although neutrophils isolated from patients with LN show evidence of *in vivo* exocytosis of specific granules, serum levels of granule cargo showed that only neutrophil elastase was elevated in LN. The ability of LN sera to stimulate HD neutrophil degranulation of secretory vesicles and specific granules, but not azurophilic granules, suggests a circulating factor in LN that, although capable of stimulating limited degranulation *in vitro*, acts as a priming agent *in vivo*. Our findings suggest the hypothesis that glomerular recruitment of primed neutrophils in LN results in enhanced intraglomerular release of toxic granule cargo on exposure to immune complex deposits, resulting in accelerated glomerular injury. This is further supported through findings from MPO immunostaining and image analysis in biopsies from patients with LN with neutrophils present within and outside of glomerular capillary loops and in the mesangial matrix. Furthermore, the majority (75%) of glomeruli in biopsies from patients with LN contain three or more neutrophils, whereas the majority (68%) of glomeruli from controls contain 0–2 neutrophils.

In conclusion, this study shows that urine excretion of neutrophil granule cargo is associated with active proliferative LN. Our data support glomerular neutrophil recruitment and subsequent degranulation as the source of that excretion. This study provides further evidence supporting a role for neutrophils in LN. Previous animal studies indicate that neutrophil granule cargoes are directly responsible for glomerular podocyte and vascular endothelial cell injury and development of proteinuria.^27,28^ This study supports the participation of neutrophil degranulation in glomerular injury in human LN and suggests that urine excretion of granule proteins is indicative of glomerular inflammation. Follow-up longitudinal studies are necessary to establish urinary excretion of *α*-1AG and other granule constituents as biomarkers of active glomerular inflammation and predictors of disease progression and response to treatment. The potential contribution of neutrophil degranulation to glomerular injury provides a target for therapeutic strategies to limit glomerular damage in LN.

## Supplementary Material

## Data Availability

All data are included in the manuscript and/or supplement.

## Supplemental Material

Supplemental Table 1. Clinical characteristics of patient cohort used for array assay.

Supplemental Table 2. Clinical characteristics of patient cohort used for ELISA validation.

Supplemental Table 3. Clinical characteristics of patients comparing active LN with remission.

Supplemental Table 4. Clinical characteristics of patient cohort used for degranulation assay.
