# Supplementary material for: Increased Urine Excretion of Neutrophil Granule Cargo in Active Proliferative Lupus Nephritis
Source: Kidney360. 2024 Jul 2;5(8):1154–66. doi: 10.34067/KID.0000000000000491 (PMC11371349; doi:10.34067/KID.0000000000000491)
Supplement: Supplementary file 1 [file kidney360-5-1154-s001.pdf]

## Supplemental Tables 1-4

**Supplemental Table 1. Clinical Characteristics of Patient Cohort used for Array Assay**

| n=10 | Age (years) | Sex (FM, M) | Self-Identified Race (Black, White, Asian) | UPCR (mg/g) | eGFR (mL/min/1.73 $m^2$) | Serum Creatinine (mg/dL) | dsDNA antibody titer (IU/mL) | C3 (mg/dL) | C4 (mg/dL) |
|---|---|---|---|---|---|---|---|---|---|
| **average** | 30 | 10, 0 | 7, 2, 1 | 3349.71 | 76.90 | 1.18 | 1154 | 63.4 | 9.3 |
| **min** | 18 | | | 734.94 | 28.00 | 0.64 | 40 | 19 | 3 |
| **max** | 44 | | | 6731.43 | 130.00 | 2.38 | 4079 | 112 | 17 |

| LN Class | Number included |
|---|---|
| III | 1 |
| IV | 5 |
| III/V | 5 |
| IV/V | 5 |

| Medication | Number of Patients |
|---|---|
| Mycophenolate mofetil | 8 |
| Prednisone | 6 |
| Cyclophosphamide | 1 |
| Tacrolimus | 1 |
| Belimumab | 2 |

## Supplemental Table 2. Clinical Characteristics of Patient Cohort used for ELISA Validation

### a. Proliferative LN Patients

**α-1AG** (n=16)

| | Age (years) | Sex (FM, M) | Self-Identified Race (Black, White, Asian) | UPCR (mg/g) | eGFR (mL/min/1.73 m$^2$) | Serum Creatinine (mg/dL) | dsDNA antibody titer (IU/mL) | C3 (mg/dL) | C4 (mg/dL) |
|---|---|---|---|---|---|---|---|---|---|
| average | 36.33 | 16, 0 | 14, 2, 0 | 1889.80 | 62.00 | 1.63 | 121.47 | 89.40 | 21.20 |
| min | 22.20 | | | 905.00 | 13.00 | 0.71 | 1.00 | 60.00 | 10.00 |
| max | 54.86 | | | 3506.73 | 120.00 | 4.27 | 739.00 | 136.00 | 38.00 |

| LN Class | Number included |
|---|---|
| III | 1 |
| IV | 5 |
| III/V | 5 |
| IV/V | 5 |

| Medication | Number of Patients |
|---|---|
| Hydroxychloroquine | 13 |
| Mycophenolate mofetil | 12 |
| Prednisone | 11 |
| Belimumab | 3 |
| Azathioprine | 2 |
| Cyclophosphamide | 1 |
| Rituximab | 1 |

**MMP-9 Lactoferrin** (n=21)

| | Age (years) | Sex (FM, M) | Self-Identified Race (Black, White, Asian) | UPCR (mg/g) | eGFR (mL/min/1.73 m$^2$) | Serum Creatinine (mg/dL) | dsDNA antibody titer (IU/mL) | C3 (mg/dL) | C4 (mg/dL) |
|---|---|---|---|---|---|---|---|---|---|
| average | 32.45 | 21, 0 | 18, 2, 1 | 2583.51 | 69.81 | 1.45 | 664.85 | 76.48 | 16.19 |
| min | 20.72 | | | 905.00 | 13.00 | 0.64 | 1.00 | 19.00 | 3.00 |
| max | 48.95 | | | 6731.43 | 130.00 | 4.27 | 4079.00 | 136.00 | 38.00 |

| LN Class | Number included |
|---|---|
| III | 3 |
| IV | 7 |
| III/V | 5 |
| IV/V | 6 |

| Medication | Number of Patients |
|---|---|
| Prednisone | 16 |
| Hydroxychloroquine | 15 |
| Mycophenolate mofetil | 14 |
| Belimumab | 5 |
| Azathioprine | 2 |
| Cyclophosphamide | 2 |
| Tacrolimus | 1 |
| Rituximab | 1 |

**Azurocidin MPO** (n=18)

| | Age (years) | Sex (FM, M) | Self-Identified Race (Black, White, Asian) | UPCR (mg/g) | eGFR (mL/min/1.73 m$^2$) | Serum Creatinine (mg/dL) | dsDNA antibody titer (IU/mL) | C3 (mg/dL) | C4 (mg/dL) |
|---|---|---|---|---|---|---|---|---|---|
| average | 33.14 | 17, 1 | 15, 2, 1 | 2340.01 | 77.44 | 1.15 | 642.88 | 85.28 | 16.94 |
| min | 20.72 | | | 734.94 | 28.00 | 0.64 | 4.00 | 19.00 | 3.00 |
| max | 48.55 | | | 6731.43 | 130.00 | 2.38 | 4079.00 | 201.00 | 47.00 |

| LN Class | Number included |
|---|---|
| III | 2 |
| IV | 5 |
| III/V | 5 |
| IV/V | 6 |

| Medication | Number of Patients |
|---|---|
| Prednisone | 14 |
| Hydroxychloroquine | 13 |
| Mycophenolate mofetil | 13 |
| Belimumab | 5 |
| Azathioprine | 1 |
| Cyclophosphamide | 2 |
| Tacrolimus | 1 |
| Rituximab | 1 |

**Neutrophil Elastase** (n= 21)

| | Age (years) | Sex (FM, M) | Self-Identified Race (Black, White, Asian) | UPCR (mg/g) | eGFR (mL/min/1.73 m$^2$) | Serum Creatinine (mg/dL) | dsDNA antibody titer (IU/mL) | C3 (mg/dL) | C4 (mg/dL) |
|---|---|---|---|---|---|---|---|---|---|
| average | 34.35 | 20, 1 | 17, 3, 1 | 2251.67 | 81.40 | 1.14 | 548.30 | 81.53 | 16.95 |
| min | 20.72 | | | 625.00 | 28.00 | 0.62 | 6.00 | 19.00 | 3.00 |
| max | 64.94 | | | 6731.43 | 145.00 | 2.38 | 4079.00 | 143.00 | 47.00 |

| LN Class | Number included |
|---|---|
| III | 3 |
| IV | 6 |
| III/V | 6 |
| IV/V | 6 |

| Medication | Number of Patients |
|---|---|
| Prednisone | 19 |
| Hydroxychloroquine | 15 |
| Mycophenolate mofetil | 17 |
| Belimumab | 6 |
| Eculizamab | 1 |
| Cyclophosphamide | 2 |
| Tacrolimus | 1 |
| Rituximab | 1 |

### b. Nephrotic Controls

**Class V LN** (n=6)

| | Age (years) | Sex (FM, M) | Self-Identified Race (Black, White, Asian) | UPCR (mg/g) | eGFR (mL/min/1.73 m$^2$) | Serum Creatinine (mg/dL) | dsDNA antibody titer (IU/mL) | C3 (mg/dL) | C4 (mg/dL) |
|---|---|---|---|---|---|---|---|---|---|
| average | 35.77 | 5, 1 | 5, 1, 0 | 6199.50 | 89.75 | 1.05 | 231.67 | 122.25 | 30.50 |
| min | 26.41 | | | 1563.00 | 54.00 | 0.54 | 1.00 | 101.00 | 17.00 |
| max | 54.56 | | | 13476.00 | 129.00 | 1.66 | 684.00 | 146.00 | 40.00 |

| Medication | Number of Patients |
|---|---|
| Mycophenolate mofetil | 5 |
| Prednisone | 5 |
| Hydroxychloroquine | 5 |
| Rituximab | 1 |

**MCD** (n=3)

| | Age (years) | Sex (FM, M) | Self-Identified Race (Black, White, Asian) | UPCR (mg/g) | eGFR (mL/min/1.73 m$^2$) | Serum Creatinine (mg/dL) |
|---|---|---|---|---|---|---|
| average | 48.60 | 1, 2 | 1, 2, 0 | 6887.33 | 96.67 | 0.89 |
| min | 39.33 | | | 5860.00 | 65.00 | 0.61 |
| max | 54.68 | | | 8899.00 | 117.00 | 1.32 |

**MGN** (n=6)

| | Age (years) | Sex (FM, M) | Self-Identified Race (Black, White, Asian) | UPCR (mg/g) | eGFR (mL/min/1.73 m$^2$) | Serum Creatinine (mg/dL) |
|---|---|---|---|---|---|---|
| average | 52.85 | 3, 3 | 0, 5, 1 | 10478.67 | 71.50 | 1.36 |
| min | 18.56 | | | 4769.00 | 26.00 | 0.43 |
| max | 71.95 | | | 14804.00 | 124.00 | 3.38 |

**Supplemental Table 3. Clinical Characteristics of Patients Comparing Active LN to Remission**

| n=15 (active LN) | Age (years) | Sex (FM, M) | Self-Identified Race (Black, White, Asian) | UPCR (mg/g) | eGFR (mL/min/1.73 $m^2$) | Serum Creatinine (mg/dL) | dsDNA antibody titer (IU/mL) | C3 (mg/dL) | C4 (mg/dL) |
|---|---|---|---|---|---|---|---|---|---|
| **average** | 35.86 | 14, 1 | 13, 2, 0 | 1360.00 | 82.29 | 1.10 | 99.20 | 105.38 | 24.08 |
| **min** | 23.58 | | | 625.00 | 36.00 | 0.62 | 4 | 53 | 6 |
| **max** | 64.94 | | | 2950.00 | 145.00 | 1.91 | 403 | 201 | 47 |

| LN Class | Number included |
|---|---|
| III | 3 |
| IV | 3 |
| III/V | 7 |
| IV/V | 2 |

| Medication | Number of Patients |
|---|---|
| Mycophenolate mofetil | 13 |
| Prednisone | 12 |
| Hydroxychloroquine | 11 |
| Belimumab | 4 |
| Eculizumab | 1 |
| Voclosporin | 1 |
| Ravulizumab | 0 |
| Rituxumab | 0 |

| n=15 (inactive LN) | Age (years) | Sex (FM, M) | Self-Identified Race (Black, White, Asian) | UPCR (mg/g) | eGFR (mL/min/1.73 $m^2$) | Serum Creatinine (mg/dL) | dsDNA antibody titer (IU/mL) | C3 (mg/dL) | C4 (mg/dL) |
|---|---|---|---|---|---|---|---|---|---|
| **average** | 36.61 | 14, 1 | 13, 2, 0 | 309.93 | 68.36 | 1.25 | 90.85 | 111.25 | 23.67 |
| **min** | 22.62 | | | 122.00 | 36.00 | 0.67 | 3 | 68 | 9 |
| **max** | 66.49 | | | 492.00 | 141.00 | 1.88 | 706 | 168 | 41 |

| LN Class | Number included |
|---|---|
| III | 3 |
| IV | 3 |
| III/V | 7 |
| IV/V | 2 |

| Medication | Number of Patients |
|---|---|
| Mycophenolate mofetil | 13 |
| Prednisone | 10 |
| Hydroxychloroquine | 12 |
| Belimumab | 2 |
| Eculizumab | 0 |
| Voclosporin | 1 |
| Ravulizumab | 1 |

**Supplemental Table 4. Clinical Characteristics of Patient Cohort used for Degranulation Assay**

| n=19 | Age (years) | Sex (FM, M) | Self-Identified Race (Black, White, Asian) | UPCR (mg/g) | eGFR (mL/min/1.73 $m^2$) | Serum Creatinine (mg/dL) | dsDNA antibody titer (IU/mL) | C3 (mg/dL) | C4 (mg/dL) |
|---|---|---|---|---|---|---|---|---|---|
| **average** | 38 | 18, 1 | 16, 3, 0 | 1120.00 | 62.85 | 1.40 | 178 | 107 | 21 |
| **min** | 22 | | | 89.00 | 19.00 | 0.70 | 1 | 54 | 7 |
| **max** | 68 | | | 5154.00 | 119.00 | 2.40 | 2815 | 201 | 44 |

| LN Class | Number included |
|---|---|
| III | 5 |
| IV | 4 |
| III/V | 5 |
| IV/V | 2 |
| V | 3 |

| Medication | Number of Patients |
|---|---|
| Mycophenolate mofetil | 17 |
| Prednisone | 15 |
| Tacrolimus | 1 |
| Belimumab | 2 |
