# Supplementary material for: Increased Urine Excretion of Neutrophil Granule Cargo in Active Proliferative Lupus Nephritis
Source: Kidney360. 2024 Jul 2;5(8):1154–66. doi: 10.34067/KID.0000000000000491 (PMC11371349; doi:10.34067/KID.0000000000000491)
Supplement: Supplementary file 2 [file kidney360-5-1154-s002.pdf]

ASN American Society of Nephrology | JASN Journal of the American Society of Nephrology | KIDNEY360 Accessing Our World From Every Angle | CJASN Clinical Journal of the American Society of Nephrology

ASN Journal Disclosure Form

As per ASN journal policy, I have disclosed any financial relationships or commitments I have held in the past 36 months as included below. I have listed my Current Employer below to indicate there is a relationship requiring disclosure. If no relationship exists, my Current Employer is not listed.

M. Barati reports the following:
Employer: University of Louisville

I understand that the information above will be published within the journal article, if accepted, and that failure to comply and/or to accurately and completely report the potential financial conflicts of interest could lead to the following: 1) Prior to publication, article rejection, or 2) Post-publication, sanctions ranging from, but not limited to, issuing a correction, reporting the inaccurate information to the authors' institution, banning authors from submitting work to ASN journals for varying lengths of time, and/or retraction of the published work.

Name: Michelle T. Barati
Manuscript ID: K360-2024-000030R1
Manuscript Title: Increased Urine Excretion of Neutrophil Granule Cargo in Active Proliferative Lupus Nephritis
Date of Completion: June 1, 2024
Disclosure Updated Date: May 20, 2024

ASN American Society of Nephrology JASN Journal of the American Society of Nephrology KIDNEY360 Accessing Our World From Every Angle CJASN Clinical Journal of the American Society of Nephrology

ASN Journal Disclosure Form

As per ASN journal policy, I have disclosed any financial relationships or commitments I have held in the past 36 months as included below. I have listed my Current Employer below to indicate there is a relationship requiring disclosure. If no relationship exists, my Current Employer is not listed.

M. Brady reports the following:
Employer: University of Louisville

I understand that the information above will be published within the journal article, if accepted, and that failure to comply and/or to accurately and completely report the potential financial conflicts of interest could lead to the following: 1) Prior to publication, article rejection, or 2) Post-publication, sanctions ranging from, but not limited to, issuing a correction, reporting the inaccurate information to the authors' institution, banning authors from submitting work to ASN journals for varying lengths of time, and/or retraction of the published work.

Name: Makayla Brady
Manuscript ID: K360-2024-000030R1
Manuscript Title: Increased Urine Excretion of Neutrophil Granule Cargo in Active Proliferative Lupus Nephritis
Date of Completion: May 31, 2024
Disclosure Updated Date: May 21, 2024

ASN American Society of Nephrology JASN Journal of the American Society of Nephrology KIDNEY360 Accessing Our World From Every Angle CJASN Clinical Journal of the American Society of Nephrology

ASN Journal Disclosure Form

As per ASN journal policy, I have disclosed any financial relationships or commitments I have held in the past 36 months as included below. I have listed my Current Employer below to indicate there is a relationship requiring disclosure. If no relationship exists, my Current Employer is not listed.

D. Caster reports the following:
Employer: University of Louisville; Consultancy: Aurinia, Cabaletta, Calliditas, Chinook (Novartis), GSK, and Travere; Ownership Interest: Individual stock holdings in Coca-Cola and Procter and Gamble; Research Funding: PI on Industry Sponsored Clinical Trials sponsored by Alexion, Chinook/Novartis, and Travere; Honoraria: Aurinia, Calliditas, Chinook/Novartis, Genentech/Roche, GSK, and Travere; Advisory or Leadership Role: Lupus Foundation of America Medical Scientific Advisory Counsel; Glomerular Diseases Editorial Board; Speakers Bureau: Aurinia, GSK, Calliditas; and Other Interests or Relationships: National Institutes of Health: NIH R01 1RO1DK126777.

I understand that the information above will be published within the journal article, if accepted, and that failure to comply and/or to accurately and completely report the potential financial conflicts of interest could lead to the following: 1) Prior to publication, article rejection, or 2) Post-publication, sanctions ranging from, but not limited to, issuing a correction, reporting the inaccurate information to the authors' institution, banning authors from submitting work to ASN journals for varying lengths of time, and/or retraction of the published work.

Name: Dawn J. Caster
Manuscript ID: K360-2024-000030R1
Manuscript Title: Increased Urine Excretion of Neutrophil Granule Cargo in Active Proliferative Lupus Nephritis
Date of Completion: May 31, 2024
Disclosure Updated Date: May 31, 2024

ASN American Society of Nephrology
JASN Journal of the American Society of Nephrology
KIDNEY360 Accessing Our World From Every Angle
CJASN Clinical Journal of the American Society of Nephrology

ASN Journal Disclosure Form

As per ASN journal policy, I have disclosed any financial relationships or commitments I have held in the past 36 months as included below. I have listed my Current Employer below to indicate there is a relationship requiring disclosure. If no relationship exists, my Current Employer is not listed.

M. Daniels reports the following:
Employer: University of Louisville

I understand that the information above will be published within the journal article, if accepted, and that failure to comply and/or to accurately and completely report the potential financial conflicts of interest could lead to the following: 1) Prior to publication, article rejection, or 2) Post-publication, sanctions ranging from, but not limited to, issuing a correction, reporting the inaccurate information to the authors' institution, banning authors from submitting work to ASN journals for varying lengths of time, and/or retraction of the published work.

Name: Michael W Daniels
Manuscript ID: K360-2024-000030R1
Manuscript Title: Increased Urine Excretion of Neutrophil Granule Cargo in Active Proliferative Lupus Nephritis
Date of Completion: June 3, 2024
Disclosure Updated Date: June 1, 2024

ASN Journal Disclosure Form

As per ASN journal policy, I have disclosed any financial relationships or commitments I have held in the past 36 months as included below. I have listed my Current Employer below to indicate there is a relationship requiring disclosure. If no relationship exists, my Current Employer is not listed.

L. Davis-Johnson has nothing to disclose.

I understand that the information above will be published within the journal article, if accepted, and that failure to comply and/or to accurately and completely report the potential financial conflicts of interest could lead to the following: 1) Prior to publication, article rejection, or 2) Post-publication, sanctions ranging from, but not limited to, issuing a correction, reporting the inaccurate information to the authors' institution, banning authors from submitting work to ASN journals for varying lengths of time, and/or retraction of the published work.

Name: Leshaia Davis-Johnson
Manuscript ID: K360-2024-000030R1
Manuscript Title: Increased Urine Excretion of Neutrophil Granule Cargo in Active Proliferative Lupus Nephritis
Date of Completion: April 23, 2024
Disclosure Updated Date: April 23, 2024

ASN Journal Disclosure Form

As per ASN journal policy, I have disclosed any financial relationships or commitments I have held in the past 36 months as included below. I have listed my Current Employer below to indicate there is a relationship requiring disclosure. If no relationship exists, my Current Employer is not listed.

A. Dubbaka reports the following:
Employer: University of Louisville School of Medicine

I understand that the information above will be published within the journal article, if accepted, and that failure to comply and/or to accurately and completely report the potential financial conflicts of interest could lead to the following: 1) Prior to publication, article rejection, or 2) Post-publication, sanctions ranging from, but not limited to, issuing a correction, reporting the inaccurate information to the authors' institution, banning authors from submitting work to ASN journals for varying lengths of time, and/or retraction of the published work.

Name: Anjali Dubbaka
Manuscript ID: K360-2024-000030R1
Manuscript Title: Increased Urine Excretion of Neutrophil Granule Cargo in Active Proliferative Lupus Nephritis
Date of Completion: April 23, 2024
Disclosure Updated Date: April 23, 2024

ASN
American Society of Nephrology
JASN
Journal of the American Society of Nephrology
KIDNEY360
Accessing Our World From Every Angle
CJASN
Clinical Journal of the American Society of Nephrology

ASN Journal Disclosure Form

As per ASN journal policy, I have disclosed any financial relationships or commitments I have held in the past 36 months as included below. I have listed my Current Employer below to indicate there is a relationship requiring disclosure. If no relationship exists, my Current Employer is not listed.

R. Lightman has nothing to disclose.

I understand that the information above will be published within the journal article, if accepted, and that failure to comply and/or to accurately and completely report the potential financial conflicts of interest could lead to the following: 1) Prior to publication, article rejection, or 2) Post-publication, sanctions ranging from, but not limited to, issuing a correction, reporting the inaccurate information to the authors' institution, banning authors from submitting work to ASN journals for varying lengths of time, and/or retraction of the published work.

Name: Rebecca Lightman
Manuscript ID: K360-2024-000030R1
Manuscript Title: Increased Urine Excretion of Neutrophil Granule Cargo in Active Proliferative Lupus Nephritis
Date of Completion: April 24, 2024
Disclosure Updated Date: April 24, 2024

ASN American Society of Nephrology JASN Journal of the American Society of Nephrology KIDNEY360 Accessing Our World From Every Angle CJASN Clinical Journal of the American Society of Nephrology

ASN Journal Disclosure Form

As per ASN journal policy, I have disclosed any financial relationships or commitments I have held in the past 36 months as included below. I have listed my Current Employer below to indicate there is a relationship requiring disclosure. If no relationship exists, my Current Employer is not listed.

C. Lynn reports the following:
Ownership Interest: Walmart INC; AT&T INC; Iron MTN INC; The Coca-Cola CO; Carnival CORP

I understand that the information above will be published within the journal article, if accepted, and that failure to comply and/or to accurately and completely report the potential financial conflicts of interest could lead to the following: 1) Prior to publication, article rejection, or 2) Post-publication, sanctions ranging from, but not limited to, issuing a correction, reporting the inaccurate information to the authors' institution, banning authors from submitting work to ASN journals for varying lengths of time, and/or retraction of the published work.

Name: Conner Wayne Lynn
Manuscript ID: K360-2024-000030R1
Manuscript Title: Increased Urine Excretion of Neutrophil Granule Cargo in Active Proliferative Lupus Nephritis
Date of Completion: May 5, 2024
Disclosure Updated Date: May 5, 2024

ASN Journal Disclosure Form

As per ASN journal policy, I have disclosed any financial relationships or commitments I have held in the past 36 months as included below. I have listed my Current Employer below to indicate there is a relationship requiring disclosure. If no relationship exists, my Current Employer is not listed.

K. McLeish reports the following:
Employer: University of Louisville; Ownership Interest: Degranin Therapeutics, LLC; Research Funding: Degranin Therapeutics; and Advisory or Leadership Role: Degranin Therapeutics.

I understand that the information above will be published within the journal article, if accepted, and that failure to comply and/or to accurately and completely report the potential financial conflicts of interest could lead to the following: 1) Prior to publication, article rejection, or 2) Post-publication, sanctions ranging from, but not limited to, issuing a correction, reporting the inaccurate information to the authors' institution, banning authors from submitting work to ASN journals for varying lengths of time, and/or retraction of the published work.

Name: Kenneth R. McLeish
Manuscript ID: K360-2024-000030R1
Manuscript Title: Increased Urine Excretion of Neutrophil Granule Cargo in Active Proliferative Lupus Nephritis
Date of Completion: April 23, 2024
Disclosure Updated Date: April 23, 2024

ASN Journal Disclosure Form

As per ASN journal policy, I have disclosed any financial relationships or commitments I have held in the past 36 months as included below. I have listed my Current Employer below to indicate there is a relationship requiring disclosure. If no relationship exists, my Current Employer is not listed.

D. Powell reports the following:
Employer: University of Louisville

I understand that the information above will be published within the journal article, if accepted, and that failure to comply and/or to accurately and completely report the potential financial conflicts of interest could lead to the following: 1) Prior to publication, article rejection, or 2) Post-publication, sanctions ranging from, but not limited to, issuing a correction, reporting the inaccurate information to the authors' institution, banning authors from submitting work to ASN journals for varying lengths of time, and/or retraction of the published work.

Name: David W. Powell
Manuscript ID: K360-2024-000030R1
Manuscript Title: Increased Urine Excretion of Neutrophil Granule Cargo in Active Proliferative Lupus Nephritis
Date of Completion: May 31, 2024
Disclosure Updated Date: April 24, 2024

ASN Journal Disclosure Form

As per ASN journal policy, I have disclosed any financial relationships or commitments I have held in the past 36 months as included below. I have listed my Current Employer below to indicate there is a relationship requiring disclosure. If no relationship exists, my Current Employer is not listed.

M. Rane has nothing to disclose.

I understand that the information above will be published within the journal article, if accepted, and that failure to comply and/or to accurately and completely report the potential financial conflicts of interest could lead to the following: 1) Prior to publication, article rejection, or 2) Post-publication, sanctions ranging from, but not limited to, issuing a correction, reporting the inaccurate information to the authors' institution, banning authors from submitting work to ASN journals for varying lengths of time, and/or retraction of the published work.

Name: Madhavi J. Rane
Manuscript ID: K360-2024-000030R1
Manuscript Title: Increased Urine Excretion of Neutrophil Granule Cargo in Active Proliferative Lupus Nephritis
Date of Completion: April 24, 2024
Disclosure Updated Date: April 24, 2024

ASN American Society of Nephrology | JASN Journal of the American Society of Nephrology | KIDNEY360 Accessing Our World From Every Angle | CJASN Clinical Journal of the American Society of Nephrology

ASN Journal Disclosure Form

As per ASN journal policy, I have disclosed any financial relationships or commitments I have held in the past 36 months as included below. I have listed my Current Employer below to indicate there is a relationship requiring disclosure. If no relationship exists, my Current Employer is not listed.

N. Shoctor reports the following:
Employer: University of Louisville

I understand that the information above will be published within the journal article, if accepted, and that failure to comply and/or to accurately and completely report the potential financial conflicts of interest could lead to the following: 1) Prior to publication, article rejection, or 2) Post-publication, sanctions ranging from, but not limited to, issuing a correction, reporting the inaccurate information to the authors' institution, banning authors from submitting work to ASN journals for varying lengths of time, and/or retraction of the published work.

Name: Nicholas A. Shoctor
Manuscript ID: K360-2024-000030R1
Manuscript Title: Increased Urine Excretion of Neutrophil Granule Cargo in Active Proliferative Lupus Nephritis
Date of Completion: April 23, 2024
Disclosure Updated Date: May 19, 2023

ASN American Society of Nephrology | JASN Journal of the American Society of Nephrology | KIDNEY360 Accessing Our World From Every Angle | CJASN Clinical Journal of the American Society of Nephrology

ASN Journal Disclosure Form

As per ASN journal policy, I have disclosed any financial relationships or commitments I have held in the past 36 months as included below. I have listed my Current Employer below to indicate there is a relationship requiring disclosure. If no relationship exists, my Current Employer is not listed.

S. Tandon has nothing to disclose.

I understand that the information above will be published within the journal article, if accepted, and that failure to comply and/or to accurately and completely report the potential financial conflicts of interest could lead to the following: 1) Prior to publication, article rejection, or 2) Post-publication, sanctions ranging from, but not limited to, issuing a correction, reporting the inaccurate information to the authors' institution, banning authors from submitting work to ASN journals for varying lengths of time, and/or retraction of the published work.

Name: Shweta Tandon
Manuscript ID: K360-2024-000030R1
Manuscript Title: Increased Urine Excretion of Neutrophil Granule Cargo in Active Proliferative Lupus Nephritis
Date of Completion: April 23, 2024
Disclosure Updated Date: May 19, 2023
